# Rivoflavin may interfere with on-line monitoring of secreted green fluorescence protein fusion proteins in *Pichia pastoris*

**DOI:** 10.1186/1475-2859-6-15

**Published:** 2007-05-18

**Authors:** Anna Surribas, David Resina, Pau Ferrer, Francisco Valero

**Affiliations:** 1 Departament d'Enginyeria Química. Escola Tècnica Superior d'Enginyeria, Universitat Autònoma de Barcelona, 08193-Bellaterra (Cerdanyola del Vallès), Spain

## Abstract

**Background:**

Together with the development of optical sensors, fluorometry is becoming an increasingly attractive tool for the monitoring of cultivation processes. In this context, the green fluorescence protein (GFP) has been proposed as a molecular reporter when fused to target proteins to study their subcellular localization or secretion behaviour. The present work evaluates the use of the GFP fusion partner for monitoring extracellular production of a *Rhizopus oryzae *lipase (ROL) in *Pichia pastoris *by means of 2D-fluorimetric techniques

**Results:**

In this study, the GFP-ROL fusion protein was successfully produced as a secreted fusion form in *P. pastoris *batch cultivations. Furthermore, both the fusion enzyme and the fluorescent protein (GFP S65T mutant) retained their biological activity. However, when multiwavelength spectrofluorometry was used for extracellular fusion protein monitoring, riboflavin appeared as a major interfering component with GFP signal. Only when riboflavin was removed by ultrafiltration from cultivation supernatants, GFP fluorescence signal linearly correlated to lipase activity

**Conclusion:**

*P. pastoris *appears to secrete/excrete significant amounts of riboflavin to the culture medium. When attempting to monitor extracellular protein production in *P. pastoris *using GFP fusions combined with multiwavelength spectrofluorimetric techniques, riboflavin may interfere with GFP fluorescence signal, thus limiting the application of some GFP variants for on-line extracellular recombinant protein quantification and monitoring purposes.

## Background

Development in bioprocess technology requires new monitoring techniques that allow a deeper understanding of the system for better bioprocess control and quality assurance. To cope with these requirements, different monitoring techniques have been developed. Among them, optical technologies present some interesting advantages since they allow non-invasive *in vivo *monitoring of the bioprocess. Reagents consumption is usually not necessary as there is no sampling or sample pre-treatment and they do offer the possibility of acquiring intracellular versatile information without interferences with cells metabolism.

Fluorometry is being increasingly used in bioprocess monitoring applications due to the development in fluorescence sensors [1-3]. The first generation of optical devices, able to acquire the fluorescence signal at one pair of excitation and emission wavelengths, have evolved into multiwavelength devices that can acquire the resultant fluorescence intensity from different fluorophores throughout a cultivation. Over the past years, multiwavelentgh fluorometry has been applied to the monitoring of different compounds in several biological systems [4,5].

In this context, the *Aequorea victoria *green fluorescence protein (GFP) has attracted a enormous interest as a molecular reporter. GFP has the advantage that its chromophore is formed in an autocatalytic cyclization that does not require a cofactor. Moreover, GFP usually maintains intact the properties of the protein which is fused to. Many applications have been developed using this protein as a reporter of gene expression, protein localization or folding [6]. Also, GFP fusions have been used for on-line monitoring of the product formation with *in-situ *methods in recombinant protein production processes [5,7,8].

The methylotrophic yeast *Pichia pastoris *has become a well-established system for heterologous protein production. The level of protein expression in *P. pastoris *depends critically on the growth conditions and, therefore, the on-line monitoring of product formation in such processes may be an attractive for faster process development and optimisation.

Recently, multiwavelength fluorometry has been applied to the monitoring of *P. pastoris *cultivation processes [5,9]. For instance, it has been shown that 2-D fluorometry can be satisfactorily applied to the monitoring of biomass and substrate but that further work should be done to improve foreign protein monitoring [9]. However, only few examples of extracellular expression of GFP or fusion GFP-proteins in *P. pastoris *have been reported, either using the *S. cerevisiae *α-mating factor signal peptide [10,11], or alternative secretion factors such as the viral secretion signal derived from the K28 virus preprotoxin [12] and the *Phaseolus vulgaris *agglutinin secretion signal [13].

The *R. oryzae *lipase (ROL) gene has been previously expressed extracellularly in *P. pastoris *under the control of the formaldehyde dehydrogenase promoter, P*FLD1 *[14,15]. In this work, the GFP protein (S65T variant) has been fused to the ROL gene and expressed in the same system in order to investigate its potential as a reporter for on-line monitoring of extracellular protein production in *P. pastoris*.

## Results and discussion

### Construction, isolation of transformants and expression studies

Previous studies on expression of GFP fusions with the lipase 1 from the yeast *Candida rugosa *in *P. pastoris *[11] indicated that GFP fusions at the N-terminus of the protein resulted in higher expression levels than when fused to the C-terminus. However, this effect has proven to be case dependent and, therefore, two fusions of the *GFP *and *ROL *genes, one with the *GFP *gene fused at the 5' end and one with the *GFP *gene fused to the 3' end of the *ROL *gene were constructed by SOE-PCR and inserted into the pPICZFLDα expression vector. *P. pastoris *transformants from the pPICZFLDαGFP-ROL and pPICZFLDαROL-GFP constructions were selected on YPD plates containing zeocin. For each construction, several clones were selected.

Five isolated clones X-33+pPICZFLDαGFP-ROL and three isolated clones X-33+ pPICZFLDαROL-GFP were precultivated in baffled shake flasks using BMS medium and methylamine as inducing substrate. Secreted recombinant ROL levels in culture supernatants were tested after 48 h. The *P. pastoris *X-33+ pPICZFLDαROL strain expressing *ROL *gene under the *FLD1 *promoter [15] was used as a reference to compare expression levels.

**Figure 1:**
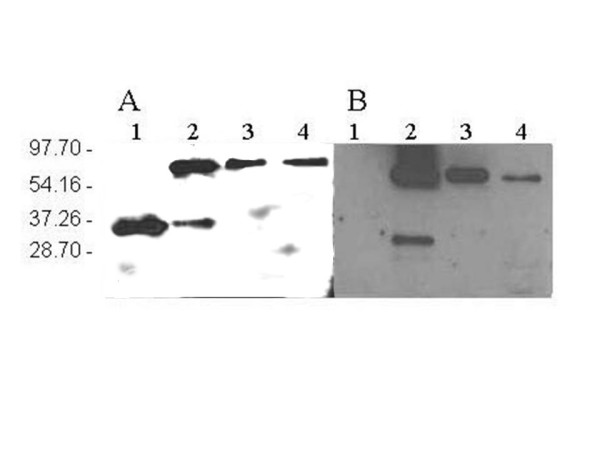
**Western blot analysis of shake flask and bioreactor batch cultivation samples**. Western blots using anti-ROL antibodies (A) and anti-GFP antibodies (B). Lane 1: ROL positive control, lane 2: culture supernatant from clone GFP-ROL 1.5.5; lane 3: culture supernatant from clone ROL-GFP 2.15.2; lane 4: culture supernatant from bioreactor culture of clone GFP-ROL 1.5.5.

The ROL-GFP transformants showed about 4-fold lower averaged specific activity levels than the GFP-ROL (0.006 ± 0.005 AU·OD^-1 ^and 0.023 ± 0.012 AU·OD^-1 ^respectively). Moreover, in all the tested clones, expression levels of the fusion protein, were at least 10-fold lower than in the cultivation with the control strain expressing ROL (0.284 ± 0.053 AU·OD^-1^). These differences in expression levels were confirmed by SDS-PAGE (data not shown). In order to assess the quality of the secreted fusion product, western blot analyses from shake flask samples of both GFP-ROL and ROL-GFP clones were performed after 66 h of cultivation (figure 1). In both cases, the analyses revealed the presence of a protein of about 60 kDa, corresponding to the fusion protein. However, culture supernatant samples from the GFP-ROL producing clones showed an additional band of approximately 30 kDa, probably corresponding to the proteolytic cleavage of the fusion product into its two components, GFP and ROL. In contrast, no degradation was observed in samples from ROL-GFP producing clones.

The X-33+pPICZFLDαGFP-ROL transformant with the highest specific activity (clone 1.5.5) was selected for further expression studies in bioreactor cultivations.

### Monitoring of extracellular GFP fluorescence levels in bioreactor batch cultivations

**Figure 2:**
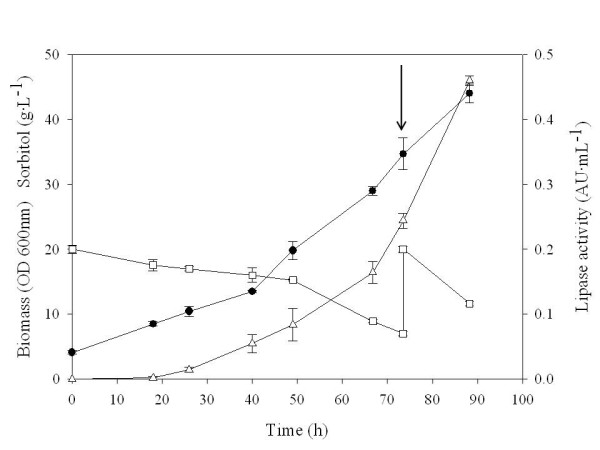
**Bioreactor batch culture of clone GFP-ROL 1.5.5**. Bioreactor batch cultivation of *P. pastoris *strain expressing the GFP-ROL fusion protein under the control of the FLD1 promoter. Sorbitol was used as the carbon source while methylamine was used both as the nitrogen source and protein inducer. Biomass (●), sorbitol concentration (□) and lipase activity (△) are depicted. Arrow indicates the addition of 15 g·L-^1 ^of sorbitol.

A batch cultivation was performed with the selected *P. pastoris *X-33+ pPICZFLDαGFP-ROL clone under bioreactor controlled conditions. A parallel control batch cultivation was performed in baffled shake flasks with the ROL-expressing X-33+pPICZFLDαROL strain. The evolution of cell density, sorbitol concentration and the lipase activity along the cultivation expressing the fusion protein is depicted in figure 2. Sorbitol was added at 73.5 hours of cultivation to further extend cell's growth phase, i.e allowing for higher product levels. Notably, in contrast to the preceding shake flask cultivations, no proteolytic degradation of the secreted fusion product was observed in the corresponding western blot analysis (figure 1, lane 4). Reduced proteolysis may be the result of better controlled cultivation conditions (pH, aeration and substrate) achieved in bioreactor cultures.

GFP fusions were evaluated as a potential tool for extracellular protein quantitative on-line monitoring in *P. pastoris*. GFP fluorescence was measured from clarified batch cultivation samples. The emission spectra for culture supernatants excited at 489 nm were analyzed. A peak appeared at 520 nm in clarified supernatants from control and GFP fusion cultivations. In both cases, this peak increased along the cultivation time. Preliminary studies (data not shown), corroborated that ROL did not have any fluorescence signal at the tested emission and excitation wavelengths, indicating that neither GFP nor ROL were responsible for the fluorescence emission signal at 520 nm observed in the supernatant of the control cultivation. This suggested that *P. pastoris *secretes a fluorescent growth-related product with a very similar excitation and emission spectra to the S65T GFP.

**Figure 3:**
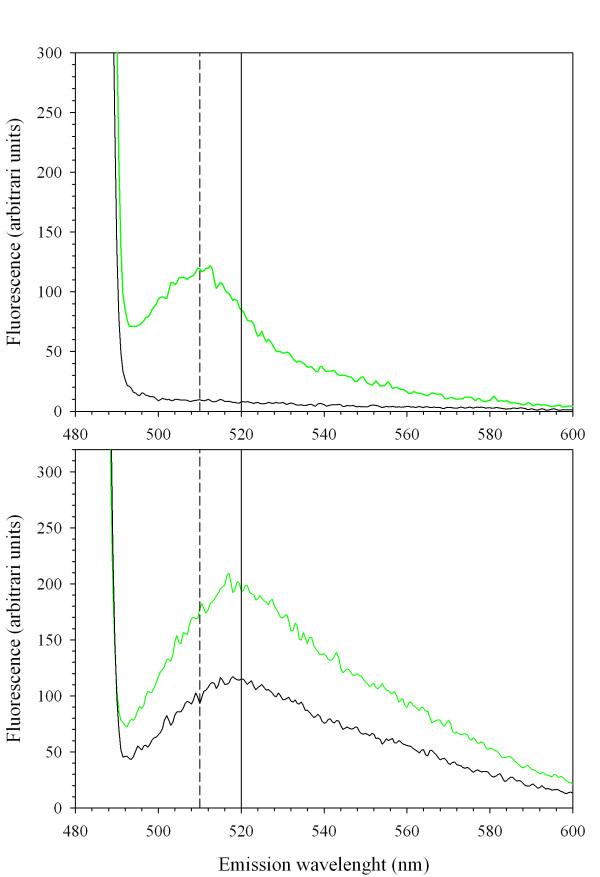
**Emission spectrum from retained and filtered culture supernatans**. Emission spectrum, given as fluorescence arbitrary units, excited at 488 nm from control strain cultivation samples (black) and GFP-fusion expressing strain cultivation samples (green line). Retained fractions (A), and corresponding filtrated fractions (B).

Recently [9], multi-wavelength on-line fluorescence measurements have been used to estimate biomass, substrate and heterologous product during a *P. pastoris *cultivation process. Riboflavin (vitamin B2), having an excitation/emission spectra of 450/530 nm, was used for biomass prediction. Riboflavin is a precursor for the synthesis of coenzymes like flavin mononucleotide (FMN) and flavin adenine dinucleotide (FAD), needed as electron acceptors by oxidoreductases. Yeasts such as *Candida famata *are industrially-employed natural overproducers of riboflavin (more than 20 g·L^-1^) [16]. Hence, our results suggest that riboflavin fluorescence could be overlapping with GFP signal in culture supernatants. Surprisingly, no significant riboflavin signal was detected in grown cells resuspended in a buffer solution. In order to remove riboflavin from culture supernatant samples, these were ultrafiltrated with a 10 kDa cut-off membrane. Ultrafiltrated and retained fractions were subsequently analyzed by spectrofluorometry. Figure 3A shows the retained fraction at 10 kDa, while figure 3B shows the filtrate fraction, presumably containing riboflavin.

As expected, the filtrate fraction excited at 489 nm showed a peak at 520 nm, most probably corresponding to riboflavin. Notably, a new peak appeared in the retained fraction excited at the same wavelength, with a maximum at 510 nm, in concordance with the GFP emission spectrum. This indicated that the extracellular GFP fluorescence could be efficiently measured in clarified culture supernatant samples after the ultrafiltration step. Also, the effect of the cultivation medium on GFP fluorescence was tested by resuspending the retained fraction from clarified batch cultivation supernatant samples in two different media: the fresh cultivation medium used for batch processes or, alternatively, 50 mM phosphate buffer, pH 7.0. The emission spectra of both samples revealed that GFP fluorescent signal is weaker under growth medium conditions (data not shown), due to the lower pH of medium (pH 5.5) with respect to phosphate buffer. This effect has also been described for the GFP S65T mutant [17].

**Figure 4:**
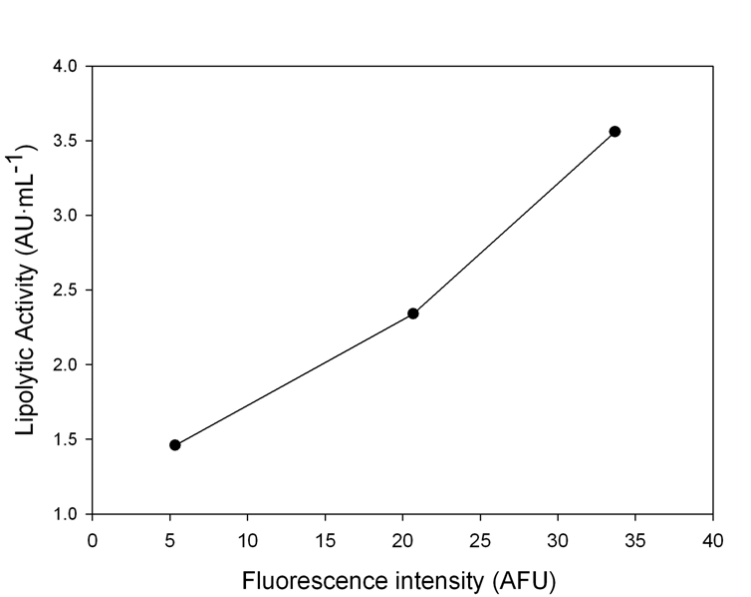
**Lipase activity versus GFP fluorescence correlations in ultrafiltrated culture supernatants**. Correlation between the measured lipolytic activity and the relative fluorescence in ultrafiltrated culture samples (●). Excitation and emission wavelengths were 489 nm and 510 nm respectively.

Importantly, extracellular GFP fluorescence and lipase activity from ultrafiltred samples were linearly correlated (figure 4), i.e. showing the potential of GFP fusions for on-line quantitative monitoring of ROL secretion in *P. pastoris *using fluorometric techniques, even in the case where secreted product titers may be low.

## Conclusion

In this work, the cloning and expression of a GFP-ROL fusion protein has been successfully achieved. A series of shake flask experiments and one batch bioreactor cultivation has been performed to test the fusion protein expression levels, as well as the feasibility to apply multiwavelength fluorometry for on-line quantitative monitoring of product secretion.

Notably, the fusion of GFP to ROL provoked a 10-fold decrease in extracellular lipase production levels in relation to the values obtained with the non-fused ROL construct. A reduction of expression levels when fusing GFP to target proteins has also been reported for other cases in *P. pastoris *[11] and *S. cerevisiae *[18]. In this context, significant levels of intracellular GFP-ROL fusion product have been detected in *P. pastoris *cells by flow cytometry and confocal microscopy techniques [19], pointing to the transport step as a major bottleneck and possibly explaining the lower extracellular product levels. Intracellular accumulation of GFP into subcellular organelles has been previously described [18,20].

When attempting to measure the extracellular GFP-ROL fusion by fluorometry, we observed that a *P. pastoris *growth-related product, probably riboflavin, overlapped the GFP signal. Only when riboflavin was removed from samples by ultrafiltration, the GFP signal could be detected in the clarified supernatants samples. Also, lipolytic activity was in good correlation with extracellular GFP fluorescence, which indicates that the GFP fusion protein was actively secreted into the medium and could be used as reporter for extracellular recombinant protein expression. Nevertheless, ultrafiltration techniques may not be readily applied to overcome such interference in on-line *in situ *fluorimetric measurements.

Overall, the efficient use of the GFP variant used in this work for on-line monitoring of extracellular recombinant protein expression in *P. pastoris *poses several limitations, which need to be considered. Other variants such as red shifted mutants or those with excitation wavelengths around 395 nm, such as GFPuv [10], might be a better option to avoid possible interferences with riboflavin or other cellular components and products. Although the decrease in the extracellular production levels would eventually hamper the application of the GFP-ROL fusion as an on-line monitoring tool, it still offers the benefit of a faster and simple detection of potential intracellular product accumulation. In addition, the use of an appropriate improved GFP mutant should avoid the riboflavin interference, i.e. allowing for both intra and extracellular product detection purposes. Altogether, these advantages can be used in the screening optimization of the ROL producing process conditions.

## Methods

### Strains

*E. coli *DH5α was used for plasmid construction and amplification. The wild-type phenotype *P. pastoris *X-33 strain (Invitrogen Co., CA, USA) was used as the host strain for the expression of a *R oryzae *lipase gene (*ROL*) fused to the *Aequoria victoria *GFP S65T variant under the transcriptional control of the *PFLD1 *promoter. The X-33/pPICZFLDα ROL derivative strain was used for comparative studies [15]. The S65T GFP mutant was used in this work due to its brighter fluorescence and its higher stability compared to the wild type form [6].

### Plasmid and strain construction

Two constructions coding for the fusion protein were performed, one coding for GFP protein fused to the ROL N-terminal end and the other fused to the ROL C-terminal. The constructions ROL-GFP and GFP-ROL were obtained by SOE-PCR [26]. The primers used for each reaction are summarized in table 1. The construction involved two steps: first, the two genes were separately amplified introducing a 12 amino acid linker between the two sequences [11,21]. The *GFP *gene was amplified from the plasmid pPICZ-GFP [11]. For the GFP-ROL fusion, the forward primer DR9 and the reverse primer DR3 were used for amplification of GFP. Primer DR9 introduced a *Xho*I restriction site at the 5' of the sequence for further cloning of the construct into the expression plasmid. Primer DR3 introduced a linker sequence codifying for 12 amino acids (marked in italics in table 1). For ROL amplification, the plasmid pPICZFLDα_ROL [14] was used as template. Primers DR4 and DR2 were used for PCR, primer DR4 contained the linker sequence (in italics) and primer DR2 a restriction site for *Not*I.

**Table 1:** Primers used for SOE-PCR

Name	Sequence	Tm °C
DR2	gtagagcggccgccaaacagcttccttcgttgatatcaaagtaactca	55.8
DR3	*aaattcaccagaaccagcagaaccagcagaacc*tttgtatagttcatccatgccatgtgtaatccc	55.8
DR4	*ccaagacgaccaagacgaccaagaccacttaaa*tctgatggtggtaaggttgttgctgctactactg	59.3
DR6	*aaattcaccagaaccagcagaaccagcagaacc*caaacagcttccttcgttgatatca	56.5
DR7	*ggttctgctggttctgctggttctggtgaattt*agtaaaggagaagaacttttcactggagt	53.0
DR9	ctctcgagaaaagagaggctgaagctgaattcatgagtaaaggagaagaacttttca	55.5
DR10	gtatctctcgagaaaagagaggctgaagctagtaaaggagaagaactt	55.5
DR11	gcggccgcttattacaaacagcttccttcgttgatatca	56.5
DR12	gtatctctcgagaaaagagaggctgaagcttctgatggtggtaaggtt	56.5
DR13	gcggccgcttattatttgtatagttcatccatgccatgtg	56.8
DR14	gtatctctcgagaaaagagaggctgaagct	61.3
DR15	gcggccgcttattatttgtatagttcatc	61.2

For ROL-GFP fusion, first DR12 and DR6 were utilized for ROL amplification. DR12 contained a *Xho*I restriction sequence and DR6 introduced a linker sequence identical to the used for GFP-ROL fusion, showed in italics (table 1). GFP was also amplified using the primers DR7 and DR13. DR7 included the linker sequence and DR13 introduced a restriction site for the *Not*I enzyme.

In a second step, PCR products containing the GFP and ROL genes were fused using the complementary linker regions as PCR primers. In the third step, primers DR14 and DR15 were added into the reaction tube to amplify the product of the SOE-PCR for the ROL-GFP fusion, DR10 and DR11 were utilized in GFP-ROL reaction.

The resulting fusion DNA fragments, GFP-ROL and ROL-GFP, both consisting of 1613 base pairs, were cut with *XhoI *and *Not*I and ligated into *Xho*I- and *Not*I-digested pPICZFLDα backbone. The DNA ligation reaction was then transformed into *E. coli *and transformants were selected on low-salt LB plates containing zeocin. Plasmidic DNA was extracted from several colonies and sequenced for confirmation of the correct fused sequences. The obtained expression vectors for each of the constructions were linearized and used for transformation of *P. pastoris *X-33 competent cells by electroporation [22]. *P. pastoris *transformants from GFP-ROL and ROL-GFP constructions were selected on YPD plates containing zeocin. For each construction four clones were selected and re-inoculated on fresh selective plates for three successive passages to ensure the isolation of pure transformant colonies.

### Media composition

*E. coli *strains were cultivated in low salt Luria broth (LB) medium, 1% (w/v) yeast extract, 1% (w/v) peptone and 0.5% (w/v) NaCl supplemented with 50 μg mL^-1 ^zeocin (Invitrogen Co., CA, USA) when necessary. *P. pastoris *strains were cultivated in YPD medium (1% (w/v) yeast extract, 2% (w/v) peptone, 2% (w/v) glucose) and 100 μg·mL^-1 ^of zeocin, when required.

Shake flask cultivations were performed using Buffered Minimal Sorbitol medium (BMS) containing 1% (w/v) sorbitol, 1.34 % (w/v) YNB without aminoacids and ammonium sulphate, 0.4 % (w/v) methylamine hydrochloride, 4·10^-5 ^% (w/v) biotin and 100 mM potassium phosphate pH 6.0.

Bioreactor batch cultivations were carried out using a mineral medium [23] with the following composition: KH_2_PO_4 _4.8 g·L^-1^, MgSO_4_·7H_2_O 1.88 g·L^-1^, CaCl_2_·2H_2_O 0.144 g·L^-1^, sorbitol 20 g·L^-1^, methylamine chloride 6 g·L^-1^, 0.1 mL·L^-1 ^of antifoam Mazu DF 7960, 1 mL·L^-1 ^of a biotin solution (400 mg·L^-1^), and 1 mL·L^-1 ^of trace salts solution (0.2 mM CuSO_4_·5H_2_O, 1.25 mM KI, 4.5 mM MnSO_4_·4H_2_O, 2 mM Na_2_MoO_4_·2H_2_O, 0.75 mM H_3_BO_3_, 17.5 mM ZnSO_4_·7H_2_O, 44.5 mM FeCl_3_·6H_2_O). The biotin and trace salts components were sterilised separately by micro filtration. The starter cultures for bioreactor cultivations were grown in YPD medium.

### Cultivation conditions

Shake flask cultivations were carried out at a working volume of 50 mL in 500 mL shake flasks. Cultivations were incubated at 30°C and 200 rpm. A starter culture of 200 mL grown on YPD medium was used to inoculate the 1.5 L bioreactor culture. Cells were harvested by centrifugation and resuspended in sterile water prior to inoculation. Bioreactor batch cultivation was performed at a working volume of 1.5 L in a 2 L bench-top bioreactor (Biostat B, Braun Biotech) at 30°C and 800 rpm. The pH of the cultivation was maintained at 5.5 by automatically adding 5 M KOH. The airflow was kept at 2 L·min-^1^, assuring a minimal dissolved oxygen concentration of 30 % throughout the cultivation time.

### Analytical procedures

Cell density was analysed by measuring the optical density at 600 nm. Sorbitol concentration was determined by a HP 1050 liquid chromatograph (Hewlett Packard) and an Aminex HPX-87H ion-exchange column from BioRad. The mobile phase was 15 mM sulphuric acid. Data were quantified by the Millenium 2.15.10 software (Waters Corporation) being 3% the obtained relative standard deviation.

Lipolytic activity determination was carried out using the Lipase colorimetric assay (kit 1821792 from Roche Diagnostics) [15] with a relative standard deviation of 10 %. Intracellular lipase activity (defined as the soluble fraction of the cell bound lipase activity) was measured from clarified supernatants from cell lysates [14].

### Fluorescence measurements

GFP fusion protein fluorescence in culture supernatant and cells samples were measured using a Perkin Elmer LS55 Spectrophotometer (PerkinElmer Ltd., Beaconsfield, UK) equipped with a xenon lamp. For intracellular GFP and cellular autofluorescence, cells were centrifuged at 12,000 rpm using a bench-top centrifuge (Biofuge Fresco Refrigerated Microcentrifuge, Heraeus, Thermo Electron LED GmbH, Langenselbold, Germany). Supernatants were removed for subsequent analyses. To avoid excitation light interferences, cells were excited at 460 nm, while supernatant were analysed at 489 nm. An excitation wavelength of 460 nm allowed for a better visualization of GFP intracellular fluorescence in intact cells, since using the optimal 480 nm excitation wavelength resulted in a partial overlap of GFP fluorescence by the excitation light. Emission fluorescence was collected over a range from 480 to 600 nm. Analyses were carried out maintaining the cuvette at 20°C. When necessary, 50 mM phosphate buffer, pH 7.0 was used to dilute samples. Once emission fluorescence scan was acquired, the maximum emission intensity was measured and used for calculations. Emission maxima were situated at 510 nm and 520 nm for the GFP and presumed riboflavin signal, respectively. Ultrafiltration units (Amicon Ultra-15 centrifugal filters, Millipore, MA, USA) with a 10 kDa cut-off membrane were utilized to separate the fusion protein from low molecular weight supernatant components.

### SDS-PAGE and Western blot analyses

Sodium dodecyl sulphate-polyacrilamide (12%) gel electrophoresis (SDS-PAGE) analyses were performed in a Mini-Protean II unit (Bio-Rad, CA, USA). Western blots were carried out after protein transference from SDS-PAGE to a nitrocellulose membrane using a Mini Trans-Blot Electrophoretic Tranfer Cell (BioRad, CA, USA) following manufacturer's instructions. For ROL detection, a mouse anti-ROL antiserum [14] was used with a dilution 1:100. For GFP detection, a mouse anti-GFP (Sigma, St. Louis, USA) was used with a dilution 1:500. Antimouse and antirabbit IgG horseradish peroxidase conjugate (Sigma, St. Louis, USA) were used to a 1:1000 dilution as secondary antibody for ROL and GFP detection, respectively. Detection was carried out with the chemiluminescent substrate SuperSignal West Pico (Pierce, IL, USA) and the signal collected by a photographic film. Prior to western blot analyses, samples from shake flask cultivations were concentrated 10-fold using the Amicon Ultra-15 centrifugal filters units (Millipore, MA, USA).

## Authors' contributions

AS and DR carried out the strains construction tasks, expression studies and fluorescence measurements. Together with, PF and FV they collaborated in the results discussion and manuscript preparation. PF and FV supervised the study and participated in the design of experiments and discussion of results.
